# Identification and characterization of a novel intronic splicing mutation in *CSF1R* ‐related leukoencephalopathy

**DOI:** 10.1111/cns.14815

**Published:** 2024-06-23

**Authors:** Yilai Han, Jinming Han, Zhen Li, Siqi Chen, Ju Liu, Ruxing Zhou, Shufang Zhao, Dawei Li, Zheng Liu, Yinan Zhao, Junwei Hao, Guoliang Chai

**Affiliations:** ^1^ Department of Neurology Xuanwu Hospital Capital Medical University, National Center for Neurological Disorders Beijing China; ^2^ Department of Neurology The First Affiliated Hospital of Zhengzhou University Zhengzhou Henan China; ^3^ Beijing Municipal Geriatric Medical Research Center Beijing China; ^4^ Key Laboratory for Neurodegenerative Diseases of Ministry of Education Beijing China; ^5^ Chinese Institutes for Medical Research Beijing China

**Keywords:** *CSF1R*‐related leukoencephalopathy, exon skipping, intronic mutation, RNA splicing

## Abstract

**Aims:**

Colony stimulating factor 1 receptor (*CSF1R*)‐related leukoencephalopathy is a rapidly progressing neurodegenerative disease caused by *CSF1R* gene mutations. This study aimed to identify and investigate the effect of a novel intronic mutation (c.1754‐3C>G) of *CSF1R* on splicing.

**Methods:**

A novel intronic mutation was identified using whole‐exome sequencing. To investigate the impact of this mutation, we employed various bioinformatics tools to analyze the transcription of the *CSF1R* gene and the three‐dimensional structure of its encoded protein. Furthermore, reverse transcription polymerase chain reaction (RT‐PCR) was performed to validate the findings.

**Results:**

A novel mutation (c.1754‐3C>G) in *CSF1R* was identified, which results in exon 13 skipping due to the disruption of the 3′ splice site consensus sequence NYAG/G. This exon skipping event was further validated in the peripheral blood of the mutation carrier through RT‐PCR and Sanger sequencing. Protein structure prediction indicated a disruption in the tyrosine kinase domain, with the truncated protein showing significant structural alterations.

**Conclusions:**

Our findings underscore the importance of intronic mis‐splicing mutations in the diagnosis and management of *CSF1R*‐related leukoencephalopathy.

## Graphical abstract

A novel intronic mutation (c.1754‐3C>G) in *CSF1R* was identified using whole‐exome sequencing, that leads to exon 13 skipping due to the disruption of the 3′ splice site consensus sequence NYAG/G. Protein structure analysis indicated a disruption in the tyrosine kinase domain, with the truncated protein showing significant structural alterations.

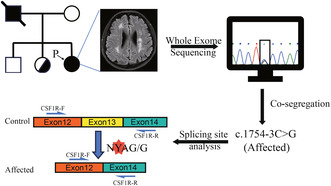

## 1 INTRODUCTION

Colony‐stimulating factor 1 receptor (*CSF1R*)‐related leukoencephalopathy is a rare progressive neurological disease caused by heterozygous mutations in the *CSF1R* gene.^1^ Approximately 300 cases have been reported related to *CSF1R* gene mutations until the year 2022,^2^, ^3^ while its genetic and phenotypic spectrum remains to be defined. The disease manifests with adult‐onset cognitive decline, psychiatric symptoms, gait disturbance, and bradykinesia. Both familial and sporadic cases have been reported, exhibiting an autosomal dominant inheritance pattern with a high penetrance.^4^ Female patients tend to exhibit clinical symptoms earlier than males.^5^ Magnetic resonance imaging (MRI) features of *CSF1R*‐related leukoencephalopathy include ventricular enlargement, cerebral atrophy, periventricular calcifications, and thinning of the corpus callosum.^6^ Typical neuropathologic findings include widespread white matter degeneration with a loss of myelin and axons, abundant neuroaxonal spheroids, and lipid‐laden and pigmented macrophages.^7^

Microglia, the resident immune cells of the central nervous system (CNS), rely on CSF1R for their survival, maintenance, and proliferation. Thus, *CSF1R*‐related leukoencephalopathy is now considered a primary CNS microgliopathy.^8^ The *CSF1R* gene encodes a ligand‐dependent tyrosine kinase receptor that belongs to the platelet‐derived growth factor (PDGF) receptor family, with CSF1 and Interleukin‐34 (IL‐34) serving as its two ligands. CSF1R contains one intracellular tyrosine kinase domain (TKD), one transmembrane domain, and five immunoglobulin‐like domains within its extracellular domain.^8^ Upon ligand binding, CSF1R undergoes dimerization and autophosphorylation, generating phosphotyrosine motifs that serve as docking sites for downstream effector pathways.^9^ Most previously reported variants lead to the inactivation of the tyrosine kinase and then reduced the CSF1R signaling.^10^ This molecular mechanism is in line with observations in patients with *CSF1R*‐related leukoencephalopathy, where microglial cell numbers and dendritic arborizations are significantly reduced.

Pathogenic *CSF1R* gene mutations may cause disease through either haploinsufficiency or dominant‐negative effects.^11^, ^12^ Most previously reported *CSF1R* mutations were located in the TKD. Although *CSF1R* missense mutations lose their kinase activity and show autosomal dominant traits, some sporadic cases can be explained by incomplete penetrance and genetic mocaicism.^13^ The haploinsufficiency or dominant negative effect cannot fully explain penetrance. When a mutated allele coexists with a highly expressed wild‐type allele, the latter is sufficient to ensure normal cell function. In both *CSF1R*‐related leukoencephalopathy patients and the knock‐in mouse model (*Csf1r* ^E631K/+^), there is a notable reduction of microglial cell numbers and dendritic arborizations.^14^ Microglia, but not neuronal reduction of CSF1R (*Cx3cr1‐Cre* ^*+/−*^; *Csf1r* ^*Flox/+*^), is sufficient to induce adult‐onset leukoencephalopathy in mice, further supporting a primary microgliopathy.

Pre‐mRNA splicing, the process of removing non‐coding introns, is critical for the expression of human genes and represents a common post‐transcriptional regulatory process in eukaryotes. Approximately 10%–62% of pathogenic single nucleotide variants affect alternative splicing processes by disrupting splicing sites.^15^ Accurate splicing requires the precise recognition of consensual donor and acceptor splice sites at the 5′ and 3′ ends of intron‐exon junctions, respectively.^16^ Maintaining the structural integrity of splicing sites is essential for ensuring proper splicing. Specifically, the canonical 5′ splice site contains the “GU” dinucleotide, which is recognized by U1 snRNP. On the other hand, the 3′ splice site, which comprises the “AG” dinucleotide and the preceding polypyrimidine tract, can also be referred to as the NYAG/G rule.^17^ It is worth noting that a minority of splicing sites may not follow the NYAG splicing rule, such as NAGNAG.^18^ These elements are crucial for splicing and are associated with U2AF1 and U2AF65, respectively.^19^, ^20^ Notably, the vast majority of pathogenic splicing mutations identified thus far are found at the 5′ ‘GU’ or 3′ ‘AG’ sites, within the ±1 or ±2 nucleotide range. This predominance may be attributed to the relatively straightforward predictability of the major impact of mutations on splicing. Apart from splicing site mutations, mutations occurring within intronic regions present a great challenge in predicting their effects on splicing.

Due to the rarity of *CSF1R*‐related leukoencephalopathy, its pathogenic mechanisms remain unclear. In this study, we identified a novel intronic mutation, c.1754‐3C > G, in the *CSF1R* gene within a family affected by *CSF1R*‐related leukoencephalopathy. This mutation disrupts the 3′ splice site NYAG/G consensus sequence, causing exon 13 skipping. Notably, while the skipping of exon 13 did not introduce a premature termination codon, it resulted in the truncation of intracellular TKD, thereby revealing a new pathogenic mechanism for splicing mutations in this disease. This discovery facilitates the monitoring of disease progression during the early stages.

## 2 METHODS

### 2.1 Subjects

A Chinese family, including one woman affected with *CSF1R*‐related leukoencephalopathy and three family members, was comprehensively evaluated by two experienced neurologists. All individuals provided informed written consent for the genetic analysis before enrollment. The Ethics Committee of Xuanwu Hospital Capital Medical University approved this study.

### 2.2 Whole‐exome sequencing

Genomic DNA was isolated from the peripheral blood of the proband and three family members. All processes were conducted according to the manufacturer's protocols. DNA purity and integrity were assessed using spectrophotometry (Thermo Fisher Scientific, Waltham, MA, USA) and agarose gel electrophoresis, respectively. Subsequently, 1 μg of DNA was broken into 250–300 bp fragments for terminal repair and joint connection using the Covaris ultrasonic instrument. Agilent SureSelect Human All Exon V6 was used to capture exons of amplified samples to prepare DNA nanospheres. Captured libraries were constructed according to the manufacturer's protocols. Sequencing was conducted using the Illumina HiSeq2000 sequencer (Illumina, San Diego, CA, USA) with a 100× sequencing coverage depth.

### 2.3 Sequencing data processing and nucleotide variation calling

The comprehensive quality control software fastp was used to filter the low‐quality reads and trim the sequencing adapter.^21^ Clean reads were aligned to reference genomes (hg19) using the BWA‐MEM software (V0.7.17) and converted into bam format files.^22^ The high credibility short variants, including single nucleotide variations (SNVs) and indels, were conducted using the GATK4 Best Practices of Germline Short Variant Discovery. The argument MarkDuplicated was used to label repeated sequences generated by sequencing. The BaseRecalibrator and ApplyBQSR commands in GATK4 were used to recalibrate the base quality score. Subsequently, the mutation detection causing both mutation and non‐mutation locations was shown in the genome variation call format (gVCF) files. Finally, the gVCF files from all samples were combined and preliminarily filtered with the screening criteria of min‐mean DP >3 and minGQ >3.

### 2.4 Variations annotation, filtering, and pathogenicity prediction

Based on the ACMG (American College of Medical Genetics) guidelines for variation classification, a variety of databases and tools were used for filtering and predicting potential pathogenicity. First, functional effects were predicted using the SnpEff toolbox. Combined annotation‐dependent depletion (CADD) is a tool for scoring the deleteriousness of SNVs and insertion/deletion variants in the human genome. A CADD score (PHREAD >10), deleterious prediction by SIFT (sorting intolerant from tolerant), or probably/possibly damaging prediction by PolyPhen was the screening criteria for missense mutations. Second, we retained the variations that lead to loss‐of‐function (LoF) variation, termination mutation (stop‐gain), termination loss (stop‐loss), frame‐shift insertions/deletions, canonical splice sites and initiation loss (start‐loss). The residual variation intolerance score (RVIS) sequenced genes according to the level of intolerance to functional genetic variants. The top 50% was performed to further filter genetic variation. Third, reported SNPs were annotated according to the dbSNP. All variants identified in the proband were screened against the 1000 Genomes Project, gnomAD database, and OMIM database. The ClinVar database was used to filter allele frequency (AF), disease citations, and other in silico attributes. The variants with minor allele frequencies (MAF ≥0.1%) were excluded.

### 2.5 Splicing site analysis

The UCSC Genome Browser (https://genome.ucsc.edu/) was used to obtain sequencing information of target fragments and transform mutant loci coordinates from hg19 to GRCh38. The splice site scores were evaluated using the Splice Prediction by Neural Network Site software (https://www.fruitfly.org/seq_tools/splice.html) to identify canonical, cryptic splicing sites between exons 12 and 14 in the *CSF1R* gene. SpliceAid2 (http://193.206.120.249/splicing_tissue.html) and Human Splicing Finder (HSF) version 2.4 (http://www.umd.be/HSF/) were utilized as web‐based bioinformatics tools to investigate the potential impact of the mutation and identified the profile changes of the exon splicing enhancer (ESE), exon splicing silencer (ESS), canonical, and cryptic splicing sites in the variable region. EX‐SKIP (https://ex‐skip.img.cas.cz/) was used to predict the effect of the mutation on the transcription process and determine if the mutation caused an exon‐skipping event. The Jalview 2.11.2.5 (https://www.jalview.org/download/) software was used for homologous nucleic acid/amino acid sequence alignment and visualization. The Open Reading Frame Finder (ORFfinder) in NCBI (https://www.ncbi.nlm.nih.gov/orffinder/) was used to identify the premature termination codons in mutated transcripts and amino acid sequences. AlphaFold2 (https://colab.research.google.com/github/sokrypton/ColabFold/blob/main/AlphaFold2.ipynb) was used to predict a 3D structure of the mutated CSF1R protein. The protein data bank (PDB) file of the wild‐type CSF1R protein was obtained from the AlphaFold Structure Database (https://alphafold.ebi.ac.uk/). The 3D structures of mutant/wild‐type CSF1R were visualized using PyMOL 2.2.3 (https://pymol.org).

### 2.6 Mutation validation by Sanger sequencing

PCR and Sanger sequencing were performed to validate identified gene mutations in peripheral genome DNA from the proband and other family members. *CSF1R*‐F: AACCCAAGTCCTCACCCCT and *CSF1R*‐R: TACCAGGTCCGCTGGAAGA were designed according to identified pathogenic mutation loci in the hg19.

### 2.7 RNA isolation, cDNA synthesis, cDNA amplification, and sequencing

To immediately stabilize intracellular RNA, fresh blood samples from the proband's sister (carrying *CSF1R* gene mutation) and healthy control were collected and preserved in the PAXgene Blood RNA Tubes (BD Bioscience). The total RNA was isolated using PAXgene Blood RNA Kits and evaluated with the Qubit 2100. Genomic DNA was removed from total RNA and then reversed transcription of RNA into cDNA using the HiScript III 1st Strand cDNA Synthesis Kit (+gDNA wiper, Vazyme). The cDNA was amplified with primers (F: CGAGAGCTATGAGGGCAACAG, R: GCTCATGATCTTCAGCTCGGAC) spanning from exons 12–14. The exon 12–14 cDNA region was amplified by PCR, and the levels of different transcripts were compared in RT‐PCR products through Sanger sequencing.

## 3 RESULTS

### 3.1 Clinical presentation and exome analysis

**FIGURE 1:**
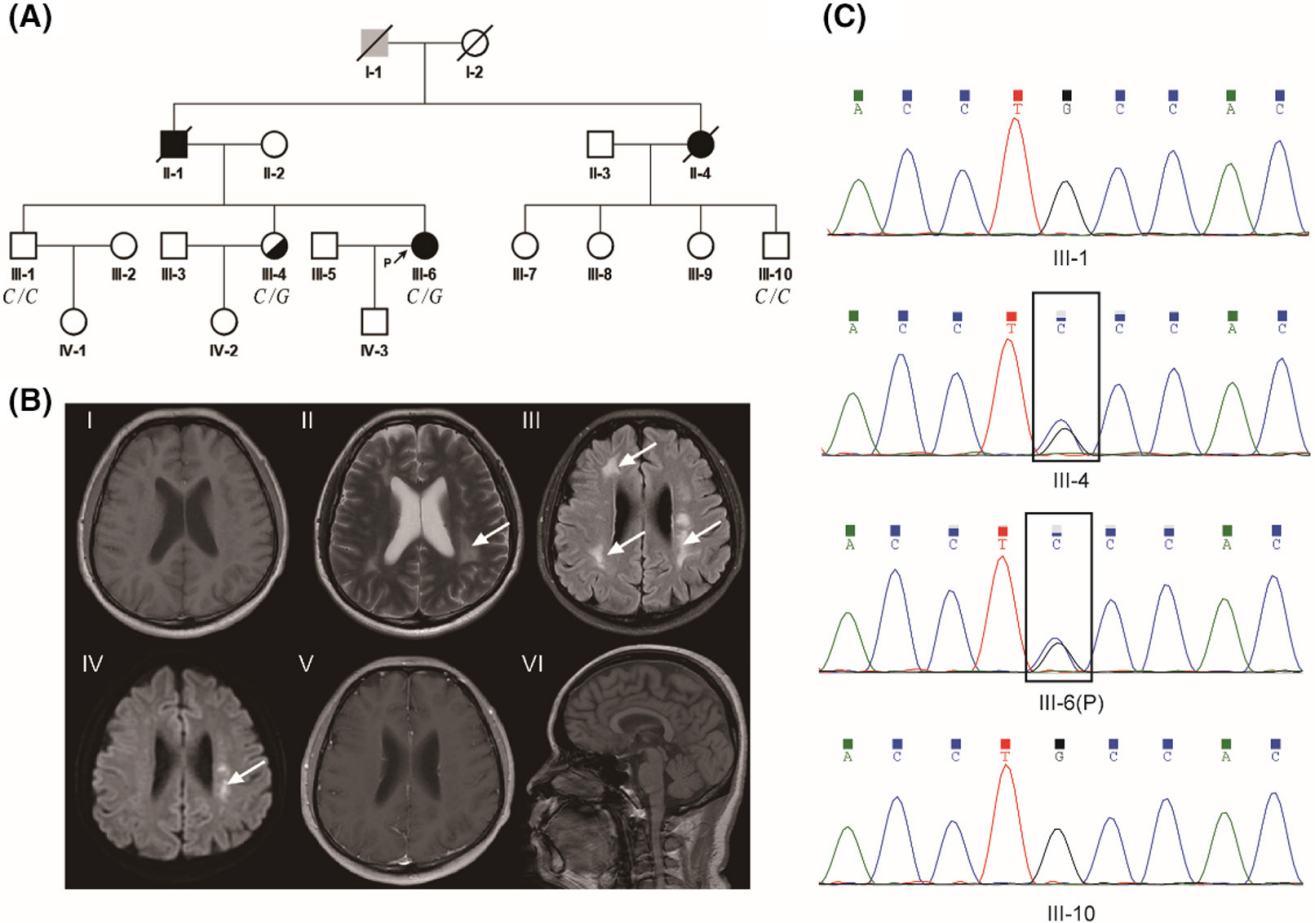
Genetic and MRI findings in a family with *CSF1R*‐related leukoencephalopathy. (A) The family pedigree spans four generations and includes 19 individuals. The proband (III‐6) is highlighted with an arrow. Solid black symbols denote affected members, while half‐filled symbols indicate carriers. (B) I: T1‐weighted axial image; II: T2‐weighted axial image; III: Fluid‐attenuated inversion recovery (FLAIR) image; IV: Diffusion‐weighted imaging (DWI) image; V: Post‐contrast T1‐weighted image; VI: T1‐weighted sagittal image. (C) Sanger sequencing chromatograms are shown for the proband (III‐6), proband's sister (III‐4), another sibling (III‐1), and a cousin (III‐10). Sequences framed in black highlight the carriers of the c.1754‐3C>G mutation in the CSF1R gene.

A 44‐year‐old Chinese woman was admitted to a local hospital due to cognitive decline and right upper extremity weakness for 7 months. She exhibited impaired social communication skills, characterized by blunted responses and unclear expressions. Approximately 2 months later, right lower extremity weakness was noted in this patient. Antiplatelet drugs and statins were administrated while her clinical symptoms did not show significant improvement. Upon admission, neurological examinations revealed tremors (right‐sided obviousness) and abnormal cerebellar signs without disability. The patient's father and aunt had experienced progressive walking difficulties and had passed away (Figure 1A). No remarkable clinical symptoms were reported in the patient's child, brother, and sister.

Routine laboratory investigations indicated the presence of hyperlipidemia, while renal and liver functions appeared to be within the normal level. Tests for arylsulphatase, metachromatic granules, and very long‐chain fatty acids yielded negative results. Cerebrospinal fluid (CSF) analysis showed normal cell counts and protein levels. Brain axial T2‐weighted and T2‐weighted fluid‐attenuated inversion recovery (FLAIR) images revealed periventricular white matter hyperintensities and abnormal signals on bilateral frontoparietal subcortex and semi‐oval centers (Figure 1B). These lesions showed low signals on T1‐weighted image, without enhancement. Additionally, some brain lesions showed diffusion restriction on diffusion‐weighted imaging (DWI).

We performed whole‐exome sequencing on the proband and identified a novel variant (c.1754‐3C>G) in the *CSF1R* gene. To analyze the significance of this mutation, we conducted comprehensive in silico analyses, including CADD, PolyPhen, and SIFT. To further validate our findings and investigate the presence of this SNV in the family, we employed Sanger sequencing (Figure 1C). Genetic testing could not be performed on her father, aunt, and grandfather since they had passed away. Among tested individuals, one sibling (III‐4) was found to carry the same *CSF1R* gene mutation, while remaining asymptomatic at the age of 49. Conversely, another sibling (III‐1) and her cousin (III‐10) were clinically unaffected and did not carry *CSF1R* gene mutations.

### 3.2 Molecular mechanisms of the splicing procedure

**FIGURE 2:**
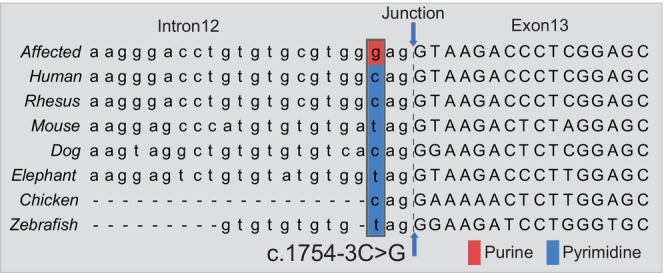
A conservative analysis is conducted on the c.1754‐3C>G mutation carrier in humans and other species. The gray frame indicates the disrupted NYAG/G conserved sequence in the mutation carrier. The red box represents Purine, and the blue signifies Pyrimidine.

The consensus sequence for the acceptor site adheres to the pattern NYAG/G (N, any nucleotide; Y, pyrimidine; “/,” inline‐exon boundary). Across various species, the mutated site within *CSF1R* follows this rule, featuring either cytosine or thymine (Figure 2). Therefore, we predicted that the c.1754‐3C>G variant may disrupt the consensus acceptor site and subsequently impact the splicing of *CSF1R*. To assess the potential impact of this mutation on RNA splicing, we conducted a series of in silico analyses. We first employed a neural network algorithm, which revealed that the nearest acceptor site of this mutation was not present in the allele (Table S1). Furthermore, we utilized HSF ver. 3.1 to evaluate the consequences of the c.1754‐3C>G mutation, including its potential effects on the distribution of canonical and cryptic splicing sites, as well as exonic splicing enhancers (ESE) and exonic splicing silencers (ESS) within the region spanning exons 12 and 14. According to the MaxEnt model, the mutation likely affects splicing, as indicated by a notable low score (6.61) at the nearest acceptor site of the mutation (Table S2). In addition to the disruption of the acceptor site, we identified two compromised ESE sites and one ESS site, which may collectively lead to a detrimental splicing event between exons 12 and 13 (Table S2). This mutation transforms the RNA binding sequence from “GCAGG/CAGGUAAGAC” to “UGGGA,” which may impact its interaction with specific RNA‐binding proteins (Figure S1). Consequently, these changes may affect the binding site of the RNA‐binding proteins (RBP), shifting from a splicing‐enhancing RBP (SF2/ASF) to a splicing‐suppressing RBP (hnRNP H1/F; Tables S3 and S4, Figure S1). In support of this, we further showed that the mutation allele had a higher chance of exon skipping than the wild‐type allele through the analysis of EX‐SKIP.

### 3.3 Identification of splicing alteration

**FIGURE 3:**
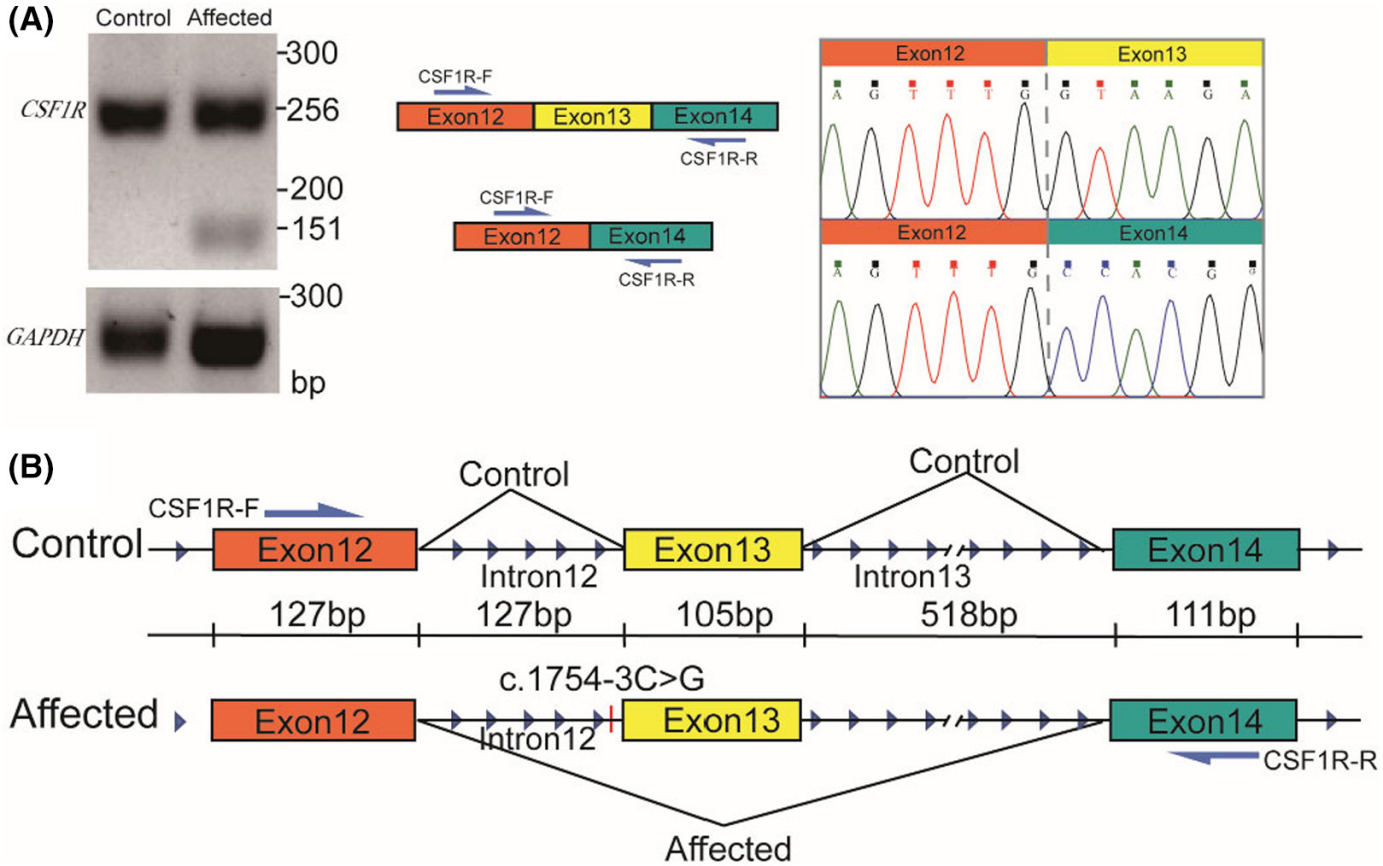
The nucleotide variation c.1754‐3C>G leads to the skipping of exon 13. (A) The RT‐PCR and Sanger results are presented for the c.1754‐3C>G mutation carrier (Affected) and a healthy control (Control). (B) The splicing patterns are depicted for Control and Affected samples between exon 12 and exon 14.

To further validate the splicing alteration, we aimed to conduct RT‐PCR from the peripheral blood cells expressing *CSF1R*. The patient had previously undergone an allogeneic hematopoietic stem cell transplant (HSCT) after diagnosis. Therefore, we decided to assess the impact of the mutation on her sister, who is an asymptomatic carrier of this mutation. We performed RT‐PCR and Sanger sequencing to analyze the splicing pattern between exons 12 and 14 in the patient's sister and a healthy control (Figures 3B and S2). Our results revealed one amplification product in the healthy control and two amplification products in the mutation carrier (Figure 3A). Upon sequencing these amplification products, we observed that the longer product (256 bp) from the carrier matched the sequence from the healthy control containing the product with exons 12, 13, and 14 (Figure 3B). In contrast, the shorter product (151 bp) contained an exon skipping event involving exon 13.

### 3.4 Impact of the patient variant on CSF1R protein

**FIGURE 4:**
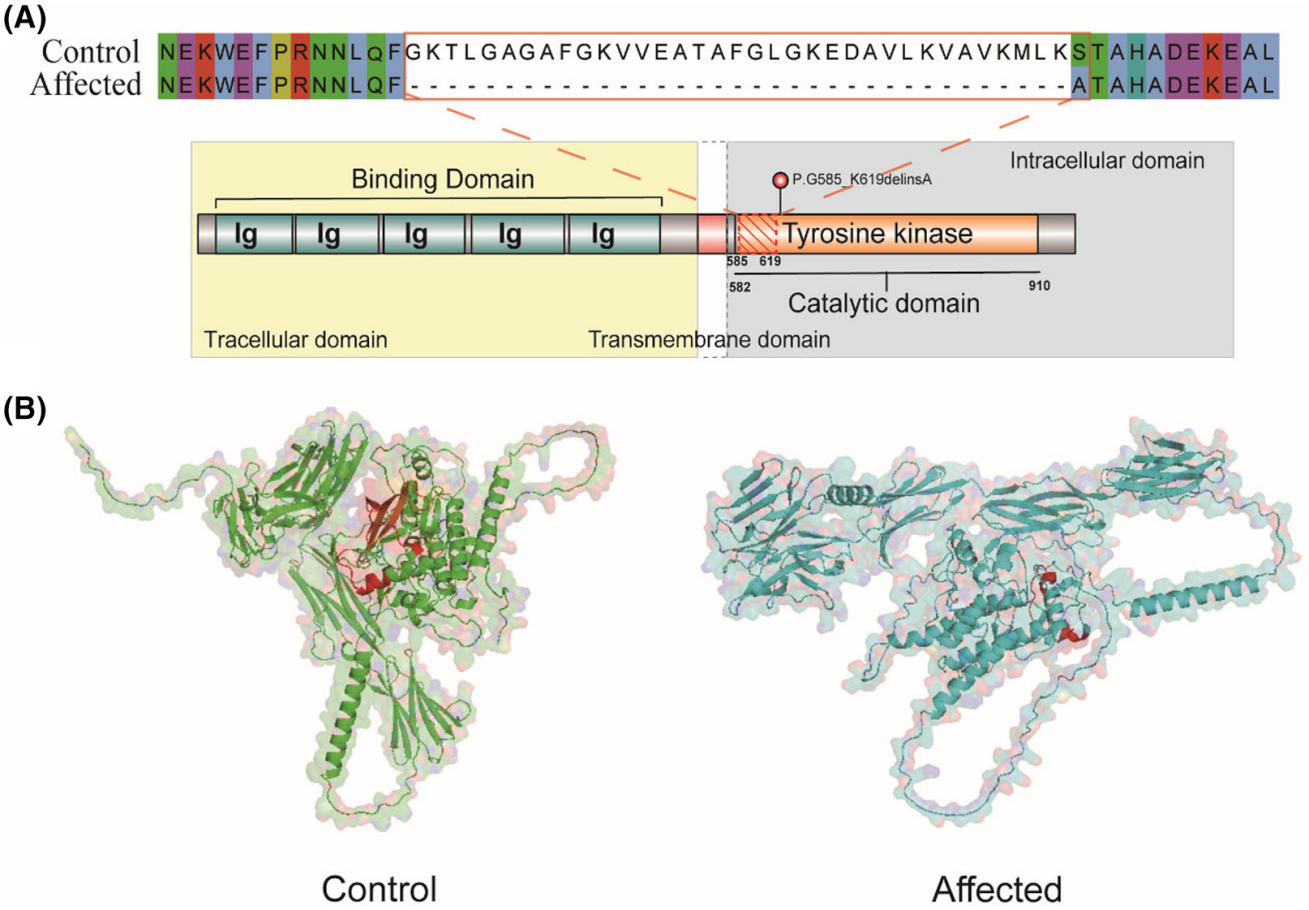
The exon skipping disrupts the TKD, resulting in a truncated protein. (A) A schematic illustration of the CSF1R protein is provided. The section highlighted with a red shadow depicts the truncated region of the TKD as a result of the mutation. The red frame showcases a comparison of amino acid sequences between the affected and control protein. (B) The 3D structure of CSF1R is displayed. In the overview of the protein structure, the red region marks the truncated section of the mutation‐affected protein.

We further evaluated the impact of exon 13 skipping on CSF1R protein. Exon 13 has a length of 105 bp, and its absence may lead to a premature stop or truncation of the protein. We performed an ORF finder analysis and our results showed that pretermination codons are not present in the mutant cDNA sequence, indicating that this mutation is likely to cause a truncated CSF1R protein (Figure 4A). Upon comparing the protein sequences of the wild‐type and truncated CSF1R, we noted that the mutant isoform lacked 35 amino acids and introduced a nonsynonymous mutation, changing lysine to alanine (P.G585_K619delinsA). A critical region situated within the conserved tyrosine kinase domain is missing, markedly impairing the enzyme's catalytic activity (Figure 4A). Subsequent analyses demonstrated that this region, inclusive of exon 13 and adjacent sequences, displays significant conservation across a variety of species. These findings highlight the indispensable role of this region in sustaining the normal function of CSF1R protein (Figure S3). We further analyzed the predicted CSF1R structure using the Alphafold model, suggesting that the affected peptide was located within a segment of the alpha helix and β‐turn angle (Figure 4B). Additionally, the structure of the truncated CSF1R isoform exhibited significant alterations. These findings collectively emphasize the potential functional consequences of this mutation on CSF1R protein.

## 4 DISCUSSION

In this study, we reported a novel intronic mis‐splicing mutation in the *CSF1R* gene associated with *CSF1R*‐related leukoencephalopathy. This mutation has not been previously documented in the literature. Interestingly, this mutation represents a non‐canonical splicing variant that does not directly affect the AG dinucleotide splicing acceptor site, and as such, might be overlooked by ACMG guidelines.^15^, ^23^ However, the c.1754‐3C>G mutation induces the skipping of exon 13 by perturbing the NYAG/G sequence rule, leading to the production of a truncated CSF1R protein within the TKD. Identifying the consequences of non‐canonical splicing variants at the RNA level is beneficial for the diagnosis of genetic diseases.

The *CSF1R* gene plays an essential role in microglial survival. Loss of the integrity of TKD within the *CSF1R* can cause dysfunctional CSF1R activity and microglial functions.^24^ *CSF1R*‐related leukoencephalopathy is characterized by a predominant involvement of microglia, classifying as microgliopathy.^10^, ^25^ Approximately 95% of previously reported *CSF1R* variants linked to leukoencephalopathy are located within the TKD, leading to the impairment of autophosphorylation. In vitro experiments have shown that cells expressing mutant *CSF1R* fail to exhibit ligand‐induced autophosphorylation.^20^, ^26^, ^27^, ^28^ Among identified mutations, over 100 missense mutations have been reported in the *CSF1R* gene.^4^, ^27^ Compared with missense mutations, splicing mutations in *CSF1R* gene were relatively rare. To date, 17 splicing site mutations have been identified related to *CSF1R*‐related leukoencephalopathy (summarized in Table 1). Among them, four mutations occur in intron 12, six in intron 18, and three in introns 13, 20, and one in 17, respectively. Similar to c.1754‐3C>G, previously reported splicing mutations affected the regions located in the kinase domain. Within the identified variation, 10 mutations adjoin the donor site, with more than six in the acceptor site and a ratio of 1.5:1, which is similar to the ratio of reported splicing mutations in HGMD (Human Gene Mutation Database).^29^, ^30^

**TABLE 1:** Summary of the CSF1R splicing site mutations.

ID	Variant	Location	Exon	Familial (F)/Sporadic (S) Disease	Trait	Reported by
1	c.1754‐10T>A	Intron 12	12_13	S	NA	Wu X et al. (2022)
**2**	**c.1754‐3C>G**	**Intron 12**	**12_13**	**F (Father, Aunt)**	**Incomplete penetrance**	**Novel Mutation**
3	c.1754‐2A>G	Intron 12	12_13	F (Brother)	Complete penetrance	Rademakers et al. (2011)
4	c.1754‐1G>C	Intron 12	12_13	F	NA	Oosterhof et al. (2019)
5	c.1858+1G>T	Intron 13	13_14	F (Mother, Brother, sister)	Complete penetrance	Yang et al. (2019)
6	c.1858+5G>A	Intron 13	13_14	F (grandma, father, uncle, brother, sister, son)	Complete penetrance	Jiang et al. (2022)
7	c. 1859‐119G>A	Intron 13	13_14	F (grandma, father, uncle, brother, sister, son)	Incomplete penetrance	Guo et al. (2019)
8	c.2320‐2A>G	Intron 17	17_18	F (Mother)	Complete penetrance	Rademakers et al. (2011)
9	c.2442+1G>T	Intron 18	18_19	S	NA	Konno et al. (2014)
10	c.2442+1G>A	Intron 18	18_19	S	NA	Lakshmanan et al. (2017)
11	c.2442+2T>C	Intron 18	18_19	F (Mother, Brother, sister)	Incomplete penetrance	YaYang et al. (2019)
12	c. 2442+2_2442+3 dupT	Intron 18	18_19	Unknown	NA	Chu et al. (2021)
13	c.2442+5G>C	Intron 18	18_19	S	NA	Konno et al. (2017)
14	c.2442+5G>A	Intron 18	18_19	F (Father)	Complete penetrance	Konno et al. (2017)
15	c.2655‐2A>G	Intron 20	20_21	Unknown	NA	Guerreiro et al. (2013)
16	c.2654+1G>A	Intron 20	20_21	F (Grandma, Mother)	Complete penetrance	Jiang et al. (2023)
17	c.2654_2654+3del	Intron 20	20_21	F (Father, Brother)	Complete penetrance	Xie et al. (2019)

*Note*: The bold text indicates the novel mutation identified in this study.

Our study identified a novel pathogenic mutation in *CSF1R* (c.1754‐3C>G) that damages the acceptor site of intron 12 and causes exon 13 skips. The absence of exon 13 disrupts the function of *CSF1R* by deleting up to 40 consecutive amino acids within the TKD. This could result in the generation of enzymatically inactive receptors, potentially causing aberrant microglial function via either haploinsufficiency or a dominant‐negative mechanism. Some previously reported *CSF1R* mutations were noted in different loci near exon 13.^8^, ^12^, ^31^ For example, Rosa et al. identified a splicing acceptor site mutation in *CSF1R* (c.1754‐2A>G) in monozygotic twins, who died of *CSF1R*‐related leukoencephalopathy at 40 and 41 years of age, respectively.^8^ The skipping of exon 13 was identified in the transcript from *CSF1R* in the mutation carriers.^8^ Nynke and colleagues reported a homozygous *CSF1R* splicing site mutation (c.1754‐1G>C) in a patient with pediatric‐onset leukoencephalopathy.^31^ This mutation can also cause exon 13 skipping. Interestingly, her parents are heterozygous carriers and did not display any clinical symptoms. Another *CSF1R* gene mutation (c.1858+1G>T) in a family with *CSF1R*‐related leukoencephalopathy was located in a splicing donor site leading to exon 13 skipping from *CSF1R* mRNA.^12^ Notably, all these previously reported mutations that cause exon 13 skipping disrupt the canonical GU‐AG splicing site. The c.1754‐1G>C and c.1754‐2A>G mutants of *CSF1R* disrupted the splicing acceptor site AG of intron 12, while the c.1858+1G>T destroyed the splicing donor site GU of intron 13.^8^, ^12^, ^31^ According to the ACMG/AMP variant classification guidelines, the disruption of highly conserved GT‐AG splicing motif (±1 or 2 splice sites) is considered as strong evidence for variants' pathogenicity.^23^ However, some SNVs introduce missense/nonsense codons, while others affect auxiliary splicing cis‐elements or generate cryptic GT‐AG dinucleotides.^19^ Therefore, some mutations may cause one part of the intron to be erroneously spliced into mature transcripts as if it were an exon. Mutation (C.1858+5G>A) disrupts the consensus sequence of the 5′ ss (from CAG/GUAAGUAU to CAG/GUAAAUAU) and breaks the complementary strand to the AUACUUACCUG sequence of U1 snRNA.^28^, ^32^ It also influences the construction of the E1 complex and causes an exon 13‐skipping event and retention of intron 12 in the *CSF1R* gene. In our study, the mutation (c.1754‐3C>G) affected the splicing procedure by breaking the NYAG/G rule near the splicing acceptor site, with the (c.1754‐3C>G) being G at intron‐3 (G at Int‐3). “G at Int‐3” is frequently observed in exons that alternatively skip in the human genome.^33^ The effect of “G at Int‐3” was further identified through cryo‐electron microscopy and isothermal titration calorimetry, showing that “G at Int‐3” decreases a binding affinity for U2AF1.^34^ The changes of NYAG/G due to the conversion of conserved pyrimidines into purines are the main causes for exon 13 skipping. The RBP binding site changing from SRSF1 to hnRNPH may cause exon 13 to skip in the *CSF1R* gene. SRSF1 binding prevents the skipping exon according to the uniprot, and the hnRNP induces the exon skipping event. The antagonistic regulation of SRSF1 and hnRNP for *COLQ* exon 16 was part of pathological processes of congenital myasthenic syndrome.^35^

Homozygous *Csf1r* knockout (*Csf1r* ^−/−^) mice exhibited global defects of brain development and then died perinatally.^36^, ^37^ Heterozygous (*Csf1r* ^+/−^) mice mimicking *CSF1R*‐related leukoencephalopathy symptoms due to *CSF1R* mutations showed cognitive and sensorimotor deficits.^38^ MRI revealed enlarged lateral ventricles and a thinner corpus callosum. *CSF1R* is mainly expressed in microglia and cortical layer V neurons, with their expression being declined in *Csf1r* ^+/−^ mice with age. Behavioral and pathological traits, along with elevated *Csf2* expression in *Csf1r* ^+/−^ mice, were replicated by microglial *Csf1r* heterozygous deletion (MCsf1r^het^) rather than neural deletion.^39^ Increased *Csf2* expression was observed in various cells in *Csf1r* ^+/−^ mice, with microglial activation evident in MCsf1r^het^ mice.^38^ The consistent rise in microglial density indicates that *CSF1R*‐related leukoencephalopathy primarily arises from microglial abnormalities. Lesions in patients with *CSF1R*‐related leukoencephalopathy are located in the frontal and parietal lobes, periventricular, and deep white matter.^6^ In the diagnostic criteria, the core imaging features are bilateral cerebral white matter lesions and corpus callosum thinning,^40^ which is associated with morphological changes in microglia.^41^ Persistent diffusion restriction can frequently be observed, which may distinguish *CSF1R*‐related leukoencephalopathy from other demyelinating diseases.

The dominant negative effect and haploinsufficiency are genetic dominance forms in some *CSF1R*‐related leukoencephalopathy families.^11^, ^27^ The former reduces the formation of active CSF‐CSF1R dimer on the cell surface through binding between the wild type and mutant CSF1R.^13^ Haploinsufficiency, where a single functional copy of a gene is insufficient for normal cellular function, has been implicated in dominantly inherited diseases.^42^ Several reports have confirmed that the severity of phenotypes is negatively correlated with the dosage of CSF1R.^43^ In patients, nonsense or frameshift mutations, as well as some splice site mutations are subjected to nonsense‐mediated mRNA decay.^44^ The haploinsufficiency can also be noted in the mouse model, with a heterozygous genotype developing *CSF1R*‐related leukoencephalopathy‐like symptoms.^38^, ^39^ In our study, the absence of exon 13 may fail to induce nonsense‐mediated decay because premature termination codons could not be identified. The production of truncated CSF1R due to c.1754‐3C>G mutation may reduce the number of active cell surface CSF1R dimer and then lead to the disease.

In our pedigree, *CSF1R*‐related leukoencephalopathy was consistent with the dominant inheritance pattern, while one carrier (the elder sister, 5 years older than the proband) displayed no clinical symptoms yet. The family declined to perform whole‐exome sequencing for their children. Incomplete penetrance has also been observed in other reported pedigrees, including those with a splicing mutation causing exon 13 skipping.^8^, ^12^, ^31^ Factors such as genetic modifiers, epigenetic modifications, or environmental exposures may potentially explain incomplete penetrance observed in this disease. Further research is required to figure out the intricate interplay of these factors in determining the clinical manifestation of *CSF1R*‐related leukoencephalopathy.

## AUTHOR CONTRIBUTIONS

YH and JH have made equal contributions to this study. They were responsible for bioinformatics analyses, and drafting and revising the manuscript. ZL verified the nucleotide mutations and exon skipping using PCR and Sanger sequencing. SC revised the figures. JL collected samples and gathered clinical data. RZ and SZ analyzed the protein structure. YZ collected the published mutation. DL and ZL gathered clinical data and provided recommendations. JH and GC spearheaded the design and coordination of the study, offered critical revisions to the manuscript, and ensured the accuracy of the data analysis.

## FUNDING INFORMATION

This study was supported by the National Key Research and Development Program of China (2021YFA1101403), the National Natural Science Foundation of China (82301526 and 82090043), Beijing Municipal Public Welfare Development and Reform Pilot Project for Medical Research Institutes (JYY2023‐7), the Project for Innovation and Development of Beijing Municipal Geriatric Medical Research Center (11000023T000002041657), the Youth Beijing Scholar (NO. 020), and the Project of Construction and Support for high‐level Innovative Teams of Beijing Municipal Institutions (BPHR20220112).

## CONFLICT OF INTEREST STATEMENT

The authors affirm that they have no conflicts of interest.

## CONSENT FOR PUBLICATION

Not applicable.

## Supporting information


Figure S1.
Figure S2.
Figure S3.

Table S1.
Table S2.
Table S3.
Table S4.

## Data Availability

The data that support the findings of this study are available from the corresponding author upon reasonable request.
