# Supplementary material for: Identification and characterization of a novel intronic splicing mutation in CSF1R ‐related leukoencephalopathy
Source: CNS Neurosci Ther. 2024 Jun 23;30(6):e14815. doi: 10.1111/cns.14815 (PMC11194178; doi:10.1111/cns.14815)
Supplement: Supplementary file 1 — Figure S1. Figure S2. Figure S3. [file CNS-30-e14815-s001.docx]

**Supplementary Figure**

# Title page

**Identification and characterization of a novel intronic splicing mutation in *CSF1R*-related leukoencephalopathy**

Yilai Han1#, Jinming Han1#, Zhen Li1, Siqi Chen1, Ju Liu2, Ruxing Zhou1, Shufang Zhao1, Dawei Li1, Zheng Liu1, Yinan Zhao1, Junwei Hao1,3,4 *, Guoliang Chai1,3,5*

1 Department of Neurology, Xuanwu Hospital Capital Medical University, Beijing, 100053, China.

2 Department of Neurology, The First Affiliated Hospital of Zhengzhou University, Zhengzhou, 450007, Henan, China.

3 Beijing Municipal Geriatric Medical Research Center, Beijing, 100053, China.

4 Key Laboratory for Neurodegenerative Diseases of Ministry of Education, Beijing, 100053, China.

5 Chinese Institutes for Medical Research, Beijing, 100069, China.

# These authors contributed equally to this work.

*Correspondence:

*Guoliang Chai,

Department of Neurology, Xuanwu Hospital Capital Medical University, Beijing 100053, China

Email: guoliang.chai@xwh.ccmu.edu.cn

*Junwei Hao, MD, PhD,

Department of Neurology, Xuanwu Hospital Capital Medical University, Beijing 100053, China.

Email: haojunwei@vip.163.com

A


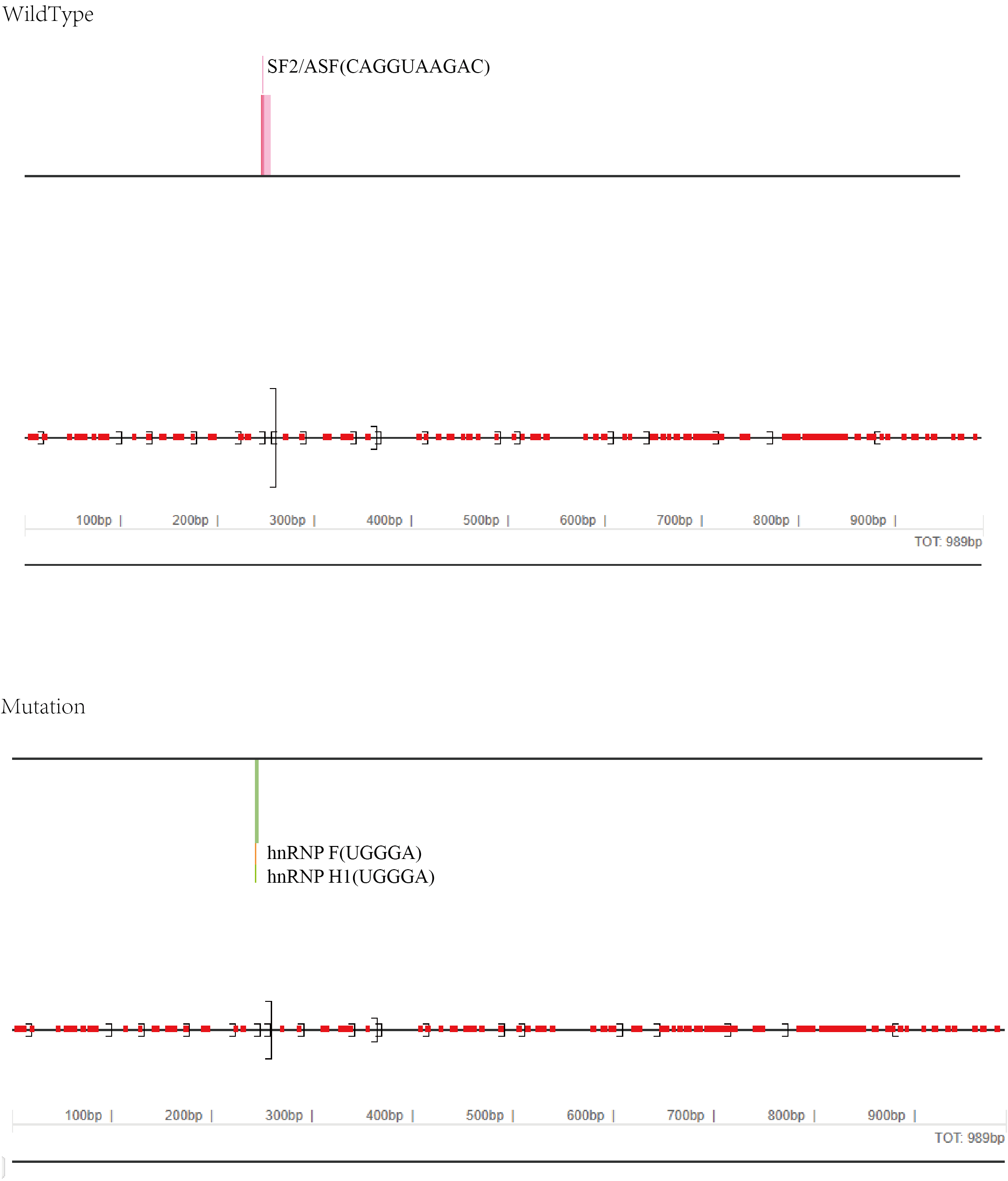


‘GAGGU’ in the control

B


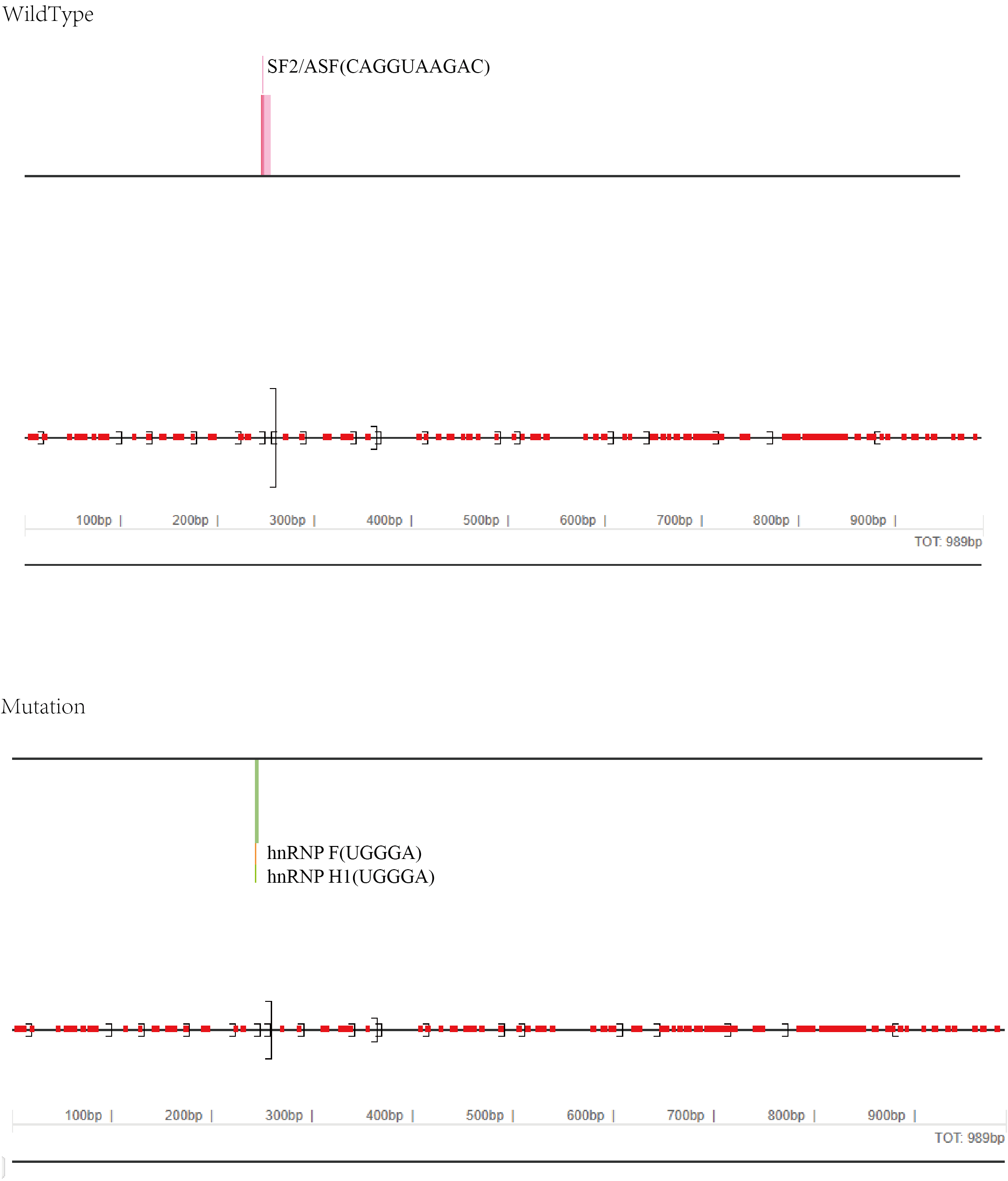


‘UGGCA’ in the control

**Figure S1.** **The *CSF1R* variation (c.1754-3C>G) inhibited the binding of ESE (SF2/ASF) and promoted the binding of ESS.** (A) The distribution of ESE and ESS in the variation region and mutation type sequences. (B) The comparison of ESE and ESS binding regions between mutated and wild-type sequences.


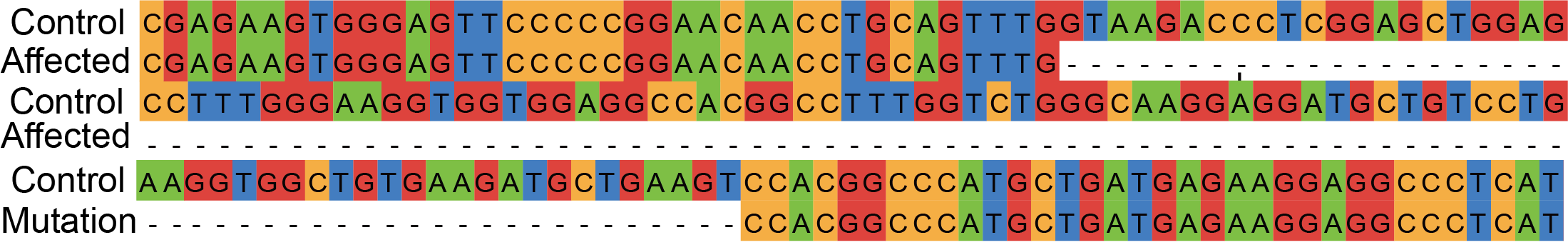


**Figure S2.** **The comparison of sequences based on Sanger sequencing around splicing regions between healthy control and mutation carrier.**


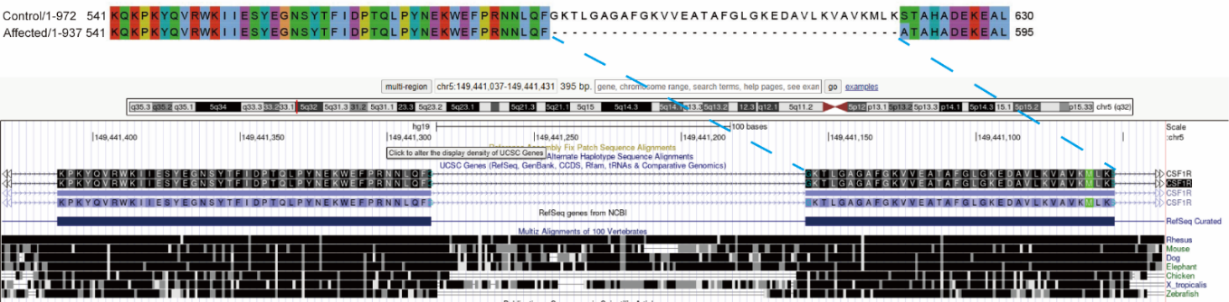


**Figure S3.** **Conservation analysis of the nucleic acid sequence corresponding to the exon 13 in *CSF1R* based on multi-species homology alignment evaluation.** The blue dashed lines indicate the exon region of abnormal splicing along with its corresponding amino acid and nucleic acid sequence.
